# KCNQ2 Gain-of-Function Mutation Presenting as Respiratory Dysfunction and Non-epileptic Myoclonus

**DOI:** 10.7759/cureus.112578

**Published:** 2026-07-13

**Authors:** Heba A Abuzayda, Raya Flayyih, Heba Abou Ali, Syed A Hosain, Junaid Khan

**Affiliations:** 1 Pediatrics, Sheikh Shakhbout Medical City (SSMC), Abu Dhabi, ARE; 2 Pediatric Neurology, Sheikh Shakhbout Medical City (SSMC), Abu Dhabi, ARE; 3 Neonatal Intensive Care Unit, Sheikh Shakhbout Medical City (SSMC), AbuDhabi, ARE

**Keywords:** channelopathy, kcnq2 gain-of-function mutation, neonatal encephalopathy, neonatal hypopnea, nonepileptic myoclonus

## Abstract

Epileptic encephalopathies are characterized by altered mental status, primarily driven by aggressive and abnormal epileptiform activities in the brain. These conditions often present with intractable seizures associated with progressive neurocognitive decline or stagnation. Mutations in the KCNQ2 gene represent one of the most common genetic causes of neonatal epileptic encephalopathies. This case report describes a rare presentation of a KCNQ2 gain-of-function mutation associated with respiratory dysfunction and non-epileptic myoclonus. This case report describes a rare presentation of a KCNQ2 gain-of-function mutation associated with respiratory dysfunction and non-epileptic myoclonus, with the patient exhibiting an uncommon phenotype of central hypopnea and non-epileptic, stimulus-sensitive myoclonus.

## Introduction

Epileptic encephalopathies are a group of severe neurological disorders characterized by impaired mental status resulting primarily from persistent epileptiform activities or other underlying etiologies [1,2]. Clinically, these disorders commonly manifest as refractory seizures accompanied by progressive neurocognitive deterioration or developmental stagnation [3]. The estimated prevalence is approximately one in 590 individuals, and although clinical improvement may occur with adequate seizure control, nearly one-third of patients remain resistant to pharmacological treatment [2,4,5]. Most epileptic encephalopathies are associated with identifiable genetic etiologies, with more than 900 causative genes reported to date; among these, variants affecting ion channel subunits constitute a major pathogenic category [6]. Pathogenic variants associated with channelopathies produce a broad spectrum of phenotypic manifestations, which are often determined by whether the underlying mutation results in gain- or loss-of-function effects [3].

Mutations in the KCNQ2 gene are among the most frequently identified genetic causes of epilepsy and neurodevelopmental delay, accounting for approximately 13% of genetic epilepsy cases [7]. The KCNQ2 gene encodes the Kv7.2 protein, one of the five subunits comprising the voltage-gated potassium channel family 7. Kv7.2 is predominantly expressed in the central nervous system, where it plays a critical role in regulating neuronal excitability by opposing the sustained membrane depolarization mediated by sodium currents [8].

The mean age at molecular diagnosis of KCNQ2-related disorders is approximately 9 months [7]. The clinical spectrum of KCNQ2-associated disease ranges from self-limiting neonatal epilepsy (SLNE) to severe developmental and epileptic encephalopathy (DEE). KCNQ2-related SLNE typically follows an autosomal dominant inheritance pattern and is distinguished from KCNQ2-DEE by a good response to antiseizure medications, normal neurological examination findings, seizure remission within the first few months of life, and generally normal neurodevelopmental outcomes. In contrast, approximately 81% of KCNQ2-DEE cases are attributed to de novo variants, most commonly resulting in loss-of-function mutations. Pathogenic copy number variants account for only about 8% of KCNQ2-DEE cases. Accordingly, genetic testing plays a critical role in establishing the diagnosis and assessing recurrence risk [9].

## Case presentation

A female neonate was delivered at 37 weeks’ gestation via emergency cesarean section due to fetal distress. She was the first child of non-consanguineous parents, with no significant antenatal or family history. Her birth weight was 2.9 kg, and Apgar scores were 7 and 9 at one and five minutes, respectively. Shortly after birth, she developed respiratory distress requiring high-flow oxygen therapy. She developed progressive respiratory dysfunction characterized by hypopnea; blood gas demonstrated respiratory acidosis, and she ultimately required mechanical ventilation at 20 hours of life.

The patient was encephalopathic with generalized hypotonia since birth and developed abnormal movements first noted at four hours of life. These episodes were characterized by brief, generalized, startle-like myoclonic jerks involving all four limbs symmetrically and were predominantly triggered by tactile and auditory stimuli. Continuous electroencephalographic monitoring demonstrated no consistent electrographic correlate of these events, suggesting they were clinically favored as nonepileptic.

Phenobarbital was initiated on the first day of life following the onset of the myoclonic jerks, and pyridoxine was added on the same day. On day 3 of life, additional therapies, including levetiracetam, pyridoxal phosphate, biotin, thiamine, and leucovorin, were introduced. A midazolam infusion was commenced on day 4, and phenytoin was subsequently added. Gradual weaning of midazolam began on day 10, with successful discontinuation achieved by day 12 of life.

**Figure 1:**
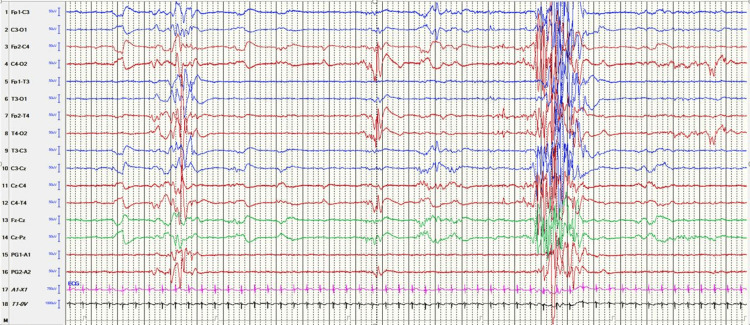
Electroencephalogram (EEG) on Day 2 of Life EEG demonstrating a burst-suppression pattern characterized by moderate- to high-amplitude polymorphic sharp and slow waves, indicative of severe cerebral dysfunction. The recording was obtained using a longitudinal bipolar montage, with a sensitivity of 7 µV/mm, a low-frequency filter of 0.5 Hz, a high-frequency filter of 70 Hz, and a paper speed of 30 mm/s.

Electroencephalography (EEG) performed on day 2 of life demonstrated a burst-suppression pattern characterized by moderate- to high-amplitude polymorphic sharp and slow wave discharges (Figure 1), consistent with severe cerebral dysfunction. The abnormal EEG background persisted despite treatment with multiple antiseizure medications. Clinically, the patient remained profoundly encephalopathic throughout her hospitalization.

**Figure 2:**
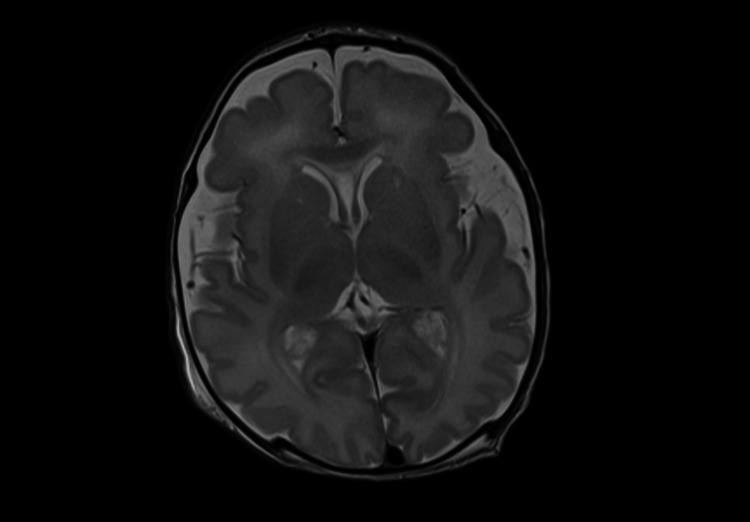
Axial T2-Weighted MRI of the Brain Performed on Day 5 of Life, Demonstrating Normal Brain Morphology With No Evidence of Structural Abnormalities, Hemorrhage, or Diffusion Restriction.

Extensive metabolic, infectious, and structural investigations were undertaken. Laboratory evaluation, including complete blood count and metabolic profile, was unremarkable (Table 1). Plasma acylcarnitine profile, plasma amino acid analysis, and cerebrospinal fluid (CSF) amino acid studies were within normal limits. CSF analysis demonstrated normal glucose and protein concentrations without pleocytosis, while CSF cultures and viral polymerase chain reaction testing were negative. Blood cultures were also negative. Brain MRI (Figure 2) revealed no structural abnormalities, and MR spectroscopy showed no abnormal metabolic peaks. Follow-up neuroimaging was not performed.

**Table 1:** Summary of Serum, Cerebrospinal Fluid, and Urine Laboratory Findings

Parameters (Unit)	Values	Referance Values
Serum		
Sodium (mmol/L)	145	135-145
Potassium (mmol/L)	3.83	3.5-5.5
Carbon Dioxide (CO_^2^_; mmol/L)	23	22- 28
Urea (mmol/l)	1.33	2.8- 8.1
Creatinine (micomol/L)	56	44-80
Serum Glucose (mmol/L)	5.3	3.9-7.8
Aspartate Aminotransferase ( IU/L)	18	<35
Alanine Transaminase (IU/L)	10	<35
Ammonia (micomol/L)	53	<100
Serum Amino Acids	Within Normal Limits	-
Acyl Carnitine Profile	Within Normal Limits	-
C-reactive Protein (mg/L)	<0.6	<5
Blood Culture	Negative for more than 48 hours	-
Hemoglobin g/L	146	145-224
Whole Blood Count (x10^9/L)	9.77	9.4-34
Platelet (x10^9/L)	319	140-400
Cerebrospinal Fluid (CSF)		
Clarity CSF	Clear	-
RBC CSF (x 10^6/L)	0	-
WBC CSF (x 10^6/L)	4	<18
Protein CSF (g/L)	1.2	<1.31
Glucose CSF (mmol/L)	4.58	>1.67
CSF Culture	Negative	-
Urine		
Urine Organic Acids	Within Normal Limits	-
Urine Culture	No Growth	-

The patient was successfully extubated to room air at two weeks of age; however, she remained encephalopathic with intermittent episodes of hypoventilation. In view of severe neonatal encephalopathy with no identifiable metabolic, infectious, or structural etiology, whole-genome sequencing was performed. Genetic analysis identified a heterozygous KCNQ2 pathogenic variant (p.R201C), confirming the diagnosis of KCNQ2-related DEE. Following the establishment of the molecular diagnosis, pyridoxine, pyridoxal phosphate, biotin, thiamine, and leucovorin were discontinued.

Despite aggressive antiseizure treatment and intensive supportive care, the patient remained profoundly neurologically impaired. She continued to experience intermittent hypoventilation episodes and required gastrostomy tube feeding because of poor sucking and swallowing. She was discharged home from the neonatal intensive care unit at seven weeks of age on phenobarbital, levetiracetam, and phenytoin. The patient died at home at nine weeks of age. The cause of death could not be determined.

## Discussion

Our patient presented with early-onset encephalopathy and stimulus-sensitive myoclonic jerks associated with a heterozygous KCNQ2 p.R201C mutation. Although this variant has been reported in multiple cases of KCNQ2-related epileptic encephalopathy, the current case exhibits distinctive clinical features that contribute to the phenotypic diversity of this genotype. To date, only 10 patients with the KCNQ2 R201C/H gain-of-function variant have been identified [10].

Consistent with previously reported KCNQ2 R201C/H cases, the EEG in our patient demonstrated a burst-suppression pattern, with subsequent follow-up recordings showing multifocal epileptiform discharges, in keeping with diffuse cerebral dysfunction. A shared clinical feature was stimulus-sensitive myoclonic events without a consistent electrographic correlate. This phenotype represents a less commonly described manifestation within the spectrum of KCNQ2-related disorders [9,10].

Neuroimaging findings further distinguish the present case. In contrast to the hypomyelination and cerebral volume loss frequently reported in other KCNQ2 R201C/H cases, the brain MRI in our patient was entirely normal. This suggests that structural abnormalities are not an obligatory feature of this genotype and may be absent early in the disease course, potentially evolving with time.

Regarding respiratory outcomes, our patient demonstrated a comparatively favorable course. Although she initially required mechanical ventilation for nine days followed by supplemental oxygen for an additional four days due to respiratory distress, she was successfully weaned and discharged in room air without ventilatory support. In contrast, many previously reported KCNQ2R201C/H cases required prolonged ventilatory support or remained oxygen-dependent for extended periods.

Therapeutic differences were also notable. In contrast to most previously reported cases, in which potassium channel openers such as retigabine (ezogabine) and/or vigabatrin were used with limited clinical benefit, our patient was managed without the use of these agents. 

KCNQ2-related DEE is generally associated with a poor neurological prognosis, particularly in patients presenting in the neonatal period with a burst-suppression EEG pattern. Emerging targeted therapies offer potential future treatment avenues. EBio 3, a small-molecule modulator, has been shown to effectively inhibit currents mediated by KCNQ2 gain-of-function variants in a non-blocking manner, providing a promising foundation for the development of precision therapies for this subgroup of KCNQ2-related disorders [11].

## Conclusions

This case illustrates a rare phenotype within the spectrum of KCNQ2-related disorders and highlights the importance of early genetic testing in infants with unexplained neonatal encephalopathy to guide management and genetic counseling. Furthermore, identifying the function of KCNQ2 pathogenic variants is essential for informing therapeutic decisions, including the potential use of targeted treatments and the avoidance of therapies that may be potentially aggravating, such as potassium channel openers in KCNQ2 gain-of-function variants.
